# Anæsthetics and Apparent Death

**Published:** 1910-06-18

**Authors:** Jas. R. Williamson

**Affiliations:** 100 Chedington Road, Upper Edmonton, London, N.


### Editor's Letter-Box.

### ANÆSTHETICS AND APPARENT DEATH.

*To the Editor of* The Hospital.

Sir,—The interesting article on "Recent Progress in Anæsthetics" in your journal of May 28 shows the necessity for the greatest precautions in the use and administration of chloroform and other anæsthetics. It may not be generally known, however, that after an attack of syncope or coma from anæsthesia, which often in appearance cannot be distinguished from death, the patient may be susceptible of recovery. Professor E. Bouchut, M.D., in his prize essay, entitled "Signes de la Mort," records several instances of persons apparently dead who recovered just in time to save them from being buried alive, and it is much to be feared that some, if not many, persons are interred whilst in a state of apparent death, due to the action of such drugs, to return to consciousness in that most hopeless, narrow prison-house underground. The following tragic incident was reported in the *New York World*, April 16, 1894: "Young Girl Buried Alive.—West Union, Ia., April 15.—A month ago the sixteen-year-old daughter of J. Tuckish, a Bohemian, living near Plotivin, Howard County, died. It appeared that the day before her death she had a tooth extracted, taking an anæsthetic, and the following morning was found, as it was supposed, dead in bed. The interment occurred the following day. A few days ago someone, in commenting on the death, said the family made a mistake in burying the girl so soon; that possibly she was not dead, and that the effect of the anæsthetic had not worn off. The parents had the body exhumed yesterday, and the glass of the coffin was found broken, the girl's hands cut and blood-stained, her hair torn out, and the corpse on its face."

It is a fact worthy of mention that if this terrible event had happened in England we should have known nothing about it, as the law forbids the exhumation of duly certified corpses, except for suspicion of poisoning or foul play of some sort, and it is not once in 50,000 burials that disinterment takes place. At a public meeting of the Association for the Prevention of Premature Burial, Dr. J. Stenson Hooker remarked on the want of unanimity in the measures taken to resuscitate patients who collapsed under the administration of chloroform and other anæsthetics. In some fatal cases, he alleged, artificial respiration was practised for an hour only, and in others for two hours. On the other hand, he (Dr. Hooker) knew of an instance where revivication ensued after eight hours' persistent artificial respiration. This seemed to indicate that hundreds of lives might be saved were efforts at resuscitation maintained for a longer period in all cases of apparent death. In the second edition of "Premature Burial, and How it may be Prevented," a case is reported by Dr. Roger G. S. Chew, of Calcutta, of a girl, eleven years of age, who was burned alive at the Burning Ghat, while in a cataleptic condition induced by the administration of chloroform; and Dr. Chew says that Surgeon Lieut.-Colonel Edward Lawrie agrees with him "that at least 90 per cent. of the chloroform deaths are preventable if proper measures are taken to resuscitate the body."

It cannot be said that cases of the kind in this country receive the attention they ought, or the fatalities would not be so numerous. The entire subject should be considered afresh in the light of evidence which has been brought out by a few indefatigable and patient scientific observers. In the meantime, if any readers of The Hospital take an interest in the important question of premature burial and its prevention, I should be pleased to send literature on the subject on receiving a stamped addressed envelope. Thanking you for your kindness,

I am, Sir, your obedient servant,

100 Chedington Road, Jas. R. Williamson.

Upper Edmonton, London, N., June 3.

---

Intimation has been made by the Secretary for Scotland that the King will be pleased to receive at St. James's Palace on June 22 addresses of loyalty from the Royal College of Physicians of Edinburgh and the Royal College of Surgeons of Edinburgh.

